# Supplementary material for: Salmonella Typhi and Salmonella Paratyphi A elaborate distinct systemic metabolite signatures during enteric fever
Source: eLife. 2014 Jun 5;3:e03100. doi: 10.7554/eLife.03100 (PMC4077204; doi:10.7554/eLife.03100)
Supplement: Supplementary file 1. — Statistically significant metabolites in pairwise comparisons. DOI: http://dx.doi.org/10.7554/eLife.03100.011 [file elife03100s001.docx]

Supplementary file

Statistically significant metabolites in pairwise comparisons

| Metabolites ^a^ | RT1^b^ | RT2^b^ | RI1^b^ | P vs. C^c^ | | | T vs. C^c^ | | | P vs. T^c^ | | |
| --- | --- | --- | --- | --- | --- | --- | --- | --- | --- | --- | --- | --- |
|  |  |  |  | p | w* | c | p | w* | c | p | w* | c |
| 1,6-anhydro-beta-glucose | 1841.2 | 2.97 | 1763.1 | - | - | - | - | - | - | - | * | P |
| 1-monohexadecanoylglycerol | 2988.7 | 3.14 | 2615.3 | *** | * | P | *** | * | T | - | - | - |
| 1-monostearoylglycerol | 3195.0 | 3.17 | 2803.2 | *** | * | P | *** | * | T | - | * | T |
| 1-palmitoyl-sn-glycero-3-phosphocholine | 3700.1 | 2.99 | 3325.7 | * | * | C | - | - | - | - | * | T |
| 2,3,4,5-tetrahydroxypentanoic acid | 1735.6 | 4.45 | 1700.0 | - | - | - | - | - | - | - | * | T |
| 2,3-butanediol | 505.1 | 2.97 | 1005.8 | - | - | - | - | - | - | - | * | P |
| 2,3-dihydroxybutenedioic acid | 1785.0 | 3.01 | 1729.1 | ** | * | P | ** | * | T | - | - | - |
| 2,4,5-trihydroxypentanoic acid | 1665.0 | 3.53 | 1658.0 | - | - | - | * | * | T | - | * | T |
| 2,4-dihydroxybutanoic acid | 1256.4 | 3.22 | 1429.6 | ** | * | P | *** | * | T | * | * | T |
| 2-aminobutyric acid | 690.1 | 2.10 | 1129.0 | * | * | P | *** | * | T | - | * | P |
| 2-ethylhexanoic acid | 825.0 | 2.78 | 1204.1 | ** | * | P | * | * | T | - | * | P |
| 2-ethyl-hydracrylic acid | 932.6 | 2.94 | 1260.8 | *** | * | P | *** | * | T | - | * | P |
| 2-hydroxy,3-methylbutanoic acid | 785.0 | 3.02 | 1182.2 | *** | * | P | *** | * | T | - | - | - |
| 2-hydroxy,4-methylpentanoic acid | 925.7 | 3.04 | 1257.3 | * | * | P | * | * | T | - | - | - |
| 2-hydroxybutanoic acid | 739.5 | 2.78 | 1156.8 | *** | * | P | *** | * | T | - | - | - |
| 2-keto-3-methylvaleric acid | 987.0 | 2.26 | 1289.1 | ** | * | P | - | * | T | - | * | P |
| 2-monopalmitin | 2959.3 | 3.07 | 2589.0 | * | * | P | - | - | - | - | - | - |
| 2-oxobutyric acid | 876.5 | 2.04 | 1230.8 | ** | * | P | *** | * | T | - | - | - |
| 2-oxoisocaproic acid | 1075.0 | 2.22 | 1334.5 | *** | * | P | * | * | T | - | * | P |
| 2-phenyl-2-hydroxypropanoic acid | 1724.9 | 2.61 | 1692.6 | * | * | P | *** | * | T | * | * | T |
| 3,4-dihydroxybutanoic acid | 1285.0 | 3.27 | 1445.0 | - | - | - | * | * | T | - | - | - |
| 3-hydroxy,3-methylbutanoic acid | 875.9 | 3.02 | 1231.0 | *** | * | P | *** | * | T | - | * | P |
| 3-hydroxybutyric acid | 805.0 | 2.82 | 1193.2 | *** | * | P | ** | * | T | - | * | P |
| 3-hydroxypentenedioic acid | 1790.8 | 3.23 | 1732.8 | ** | * | P | *** | * | T | - | * | T |
| 3-hydroxypropanoic acid | 810.0 | 2.62 | 1195.9 | * | * | P | - | - | - | - | - | - |
| 3-hydroxyvaleric acid | 947.1 | 2.91 | 1268.4 | ** | * | P | *** | * | T | - | * | P |
| 3-methylglutaconic acid | 1500.0 | 2.35 | 1562.4 | - | - | - | * | * | C | - | - | - |
| 4-hydroxybutanoic acid | 980.1 | 2.75 | 1285.8 | - | * | P | - | - | - | - | * | P |
| 4-hydroxyphenylacetic acid | 1860.9 | 2.41 | 1774.7 | * | * | C | - | - | - | - | * | T |
| 4-methyl-pentanoic acid | 627.6 | 2.40 | 1092.8 | * | * | P | - | - | - | * | * | P |
| Adenosine-5-monophosphate | 3665.0 | 2.63 | 3286.3 | *** | * | P | *** | * | T | - | - | - |
| Alanine | 677.5 | 2.88 | 1122.4 | - | - | - | - | - | - | - | * | P |
| Alpha-linolenic acid | 2666.5 | 2.64 | 2343.3 | - | - | - | - | - | - | - | * | P |
| Alpha-tocopherol | 3680.0 | 2.93 | 3303.3 | - | - | - | * | * | T | - | * | T |
| Aminomalonic acid | 1454.0 | 2.80 | 1537.1 | ** | * | C | * | * | C | - | - | - |
| Anthranilic acid | 1845.0 | 2.35 | 1765.1 | - | - | - | - | - | - | - | * | P |
| Arginine | 2034.8 | 2.90 | 1885.6 | - | - | - | *** | * | C | - | * | P |
| Asparagine | 1840.6 | 2.66 | 1762.6 | - | - | - | - | * | T | - | * | T |
| Benzoic acid | 1190.1 | 2.19 | 1393.4 | - | - | - | * | * | T | - | * | T |
| Beta-alanine | 1260.0 | 3.44 | 1431.6 | *** | * | P | *** | * | T | - | * | T |
| Campesterol | 3771.4 | 3.46 | 3405.4 | * | * | P | ** | * | T | - | * | T |
| Caprylic acid | 1047.3 | 2.72 | 1320.5 | ** | * | P | * | * | T | - | * | P |
| Cholesterol | 3670.3 | 3.37 | 3293.0 | *** | * | C | ** | * | C | - | * | T |
| cis-10-heptadecenoic acid | 2474.9 | 2.85 | 2194.6 | * | * | P | ** | * | T | - | * | T |
| cis-5-dodecenoic acid | 1740.0 | 2.77 | 1701.7 | *** | * | P | *** | * | T | - | - | - |
| Citric acid | 1977.5 | 3.20 | 1848.8 | - | - | - | - | - | - | - | * | T |
| Creatinine | 1614.0 | 2.88 | 1627.6 | *** | * | P | ** | * | T | - | * | T |
| Cysteine | 1580.0 | 2.96 | 1607.6 | - | - | - | * | * | T | * | * | T |
| Cystine | 2690.0 | 2.99 | 2362.4 | ** | * | P | - | - | - | - | * | P |
| Decanoic acid | 1406.9 | 2.85 | 1511.0 | *** | * | P | *** | * | T | - | * | T |
| Docosahexaenoic acid | 3135.0 | 2.52 | 2747.7 | *** | * | P | *** | * | T | - | * | T |
| Eicosadienoic acid | 2859.8 | 2.86 | 2502.5 | - | - | - | - | - | - | - | * | T |
| Elaidic acd/Oleic acid | 2603.8 | 2.88 | 2293.6 | ** | * | P | ** | * | T | - | - | - |
| Erythritol/Threitol | 1295.0 | 4.42 | 1451.0 | - | - | - | * | * | T | - | * | T |
| Ethanolamine | 880.0 | 3.88 | 1233.6 | ** | * | P | - | - | - | ** | * | P |
| Fructose | 1799.7 | 4.50 | 1738.9 | * | * | P | ** | * | T | - | - | - |
| Fucose | 1695.0 | 4.27 | 1676.0 | * | * | P | *** | * | T | - | * | T |
| Galacturonic acid | 2055.0 | 4.19 | 1899.5 | *** | * | C | ** | * | C | - | - | - |
| Gluconic acid | 1985.0 | 0.16 | 1851.7 | * | * | P | *** | * | T | * | * | T |
| Gluconic acid lactone | 2079.6 | 3.24 | 1915.2 | - | - | - | ** | * | C | - | - | - |
| Glucuronic acid | 2045.0 | 4.05 | 1892.9 | *** | * | C | *** | * | C | - | * | T |
| Glutamine | 1784.7 | 2.13 | 1728.4 | *** | * | P | ** | * | T | - | - | - |
| Glyceric acid | 1105.0 | 3.16 | 1350.3 | ** | * | P | ** | * | T | - | - | - |
| Glyceric acid-2-phosphate | 1999.9 | 2.74 | 1863.0 | *** | * | P | *** | * | T | - | - | - |
| Glyceric acid-3-phosphate | 2040.0 | 2.75 | 1888.9 | *** | * | P | *** | * | T | - | * | T |
| Glycerol | 918.3 | 3.66 | 1253.7 | ** | * | P | - | - | - | - | * | P |
| Glycerol-3-phosphate | 1915.0 | 2.99 | 1808.4 | * | * | P | ** | * | T | - | - | - |
| Glycine | 1024.9 | 3.44 | 1309.4 | - | - | - | - | - | - | * | * | P |
| Heptadecanoic acid | 2473.1 | 2.98 | 2193.4 | * | * | P | ** | * | T | - | * | T |
| Heptanoic acid | 865.9 | 2.60 | 1225.5 | - | - | - | - | - | - | - | * | P |
| Hexadecanoic acid methyl ester | 2265.0 | 2.59 | 2041.1 | ** | * | C | *** | * | C | - | * | P |
| Histidine | 2391.1 | 2.23 | 2132.1 | *** | * | C | ** | * | C | - | - | - |
| Homocysteine | 1744.7 | 2.96 | 1704.6 | *** | * | P | ** | * | T | - | - | - |
| Hydroxylamine | 612.1 | 3.49 | 1082.6 | * | * | P | - | - | - | - | * | P |
| Hydroxyphenyllactic acid | 2165.2 | 2.87 | 1972.1 | ** | * | P | ** | * | T | - | - | - |
| Hydroxyproline | 1450.0 | 3.40 | 1535.2 | - | - | - | - | - | - | ** | * | T |
| Hypoxanthine | 2290.0 | 2.06 | 2058.6 | ** | * | P | ** | * | T | - | - | - |
| Indole-3-acetic acid | 2520.0 | 2.11 | 2228.5 | - | - | - | - | - | - | - | * | T |
| Isocitric acid | 2014.0 | 2.95 | 1872.3 | - | * | P | * | * | T | - | - | - |
| Isoleucine | 1012.2 | 3.32 | 1302.9 | * | * | P | - | - | - | * | * | P |
| Lactic acid | 619.7 | 2.63 | 1087.4 | - | - | - | - | - | - | - | * | P |
| Lauric acid | 1745.0 | 2.91 | 1704.8 | ** | * | P | ** | * | T | - | - | - |
| Leucine | 970.0 | 3.28 | 1280.7 | *** | * | P | *** | * | T | - | * | P |
| Linoleic acid | 2625.1 | 2.77 | 2310.3 | - | - | - | ** | * | T | - | * | T |
| Lysine | 2023.4 | 3.91 | 1878.9 | *** | * | C | *** | * | C | - | - | - |
| Malic acid | 1448.0 | 2.95 | 1533.9 | ** | * | P | *** | * | T | - | * | T |
| Methionine | 1580.0 | 2.68 | 1607.5 | - | - | - | - | - | - | - | * | T |
| Methylcysteine | 1360.0 | 2.07 | 1484.9 | *** | * | C | ** | * | C | - | - | - |
| Monomethylphosphate | 1005.1 | 2.19 | 1298.5 | - | - | - | - | - | - | * | * | P |
| Monosaccharide_131 | 1760.0 | 0.18 | 1712.2 | - | * | P | * | * | T | - | - | - |
| Monosaccharide_132 | 2114.3 | 4.10 | 1938.9 | *** | * | C | *** | * | C | - | - | - |
| Monosaccharide_133 | 1904.9 | 4.66 | 1802.9 | - | - | - | - | - | - | - | * | T |
| Monosaccharide_136 | 2460.0 | 4.04 | 2184.5 | *** | * | C | * | * | C | - | * | T |
| Monosaccharide_137 | 1622.5 | 4.87 | 1633.8 | ** | * | C | - | - | - | ** | * | T |
| Monosaccharide_138 | 1669.9 | 4.12 | 1661.2 | - | - | - | - | - | - | - | * | T |
| Monosaccharide_139 | 2070.3 | 4.38 | 1909.8 | - | - | - | - | - | - | - | * | T |
| Monosaccharide_140 | 1960.3 | 4.71 | 1838.7 | * | * | C | * | * | C | - | - | - |
| Monosaccharide_141 | 1900.1 | 4.47 | 1799.8 | * | * | C | - | - | - | - | - | - |
| Monosaccharide_142 | 1630.6 | 3.88 | 1637.9 | - | - | - | - | - | - | - | * | T |
| Monosaccharide_833 | 1887.9 | 4.69 | 1792.5 | - | - | - | * | * | T | - | * | T |
| Monosaccharide_936 | 2037.3 | 3.70 | 1887.7 | * | * | C | - | - | - | - | * | T |
| myo-Inositol-1-phosphate | 2640.0 | 4.42 | 2323.5 | ** | * | C | * | * | C | - | * | T |
| Myristoleic acid | 2073.6 | 2.80 | 1910.9 | *** | * | P | *** | * | T | - | - | - |
| N-acetyl glucosamine/N-acetyl mannosamine | 2368.9 | 3.24 | 2116.4 | * | * | P | ** | * | T | - | - | - |
| N-acetyl-alanine | 1280.0 | 1.96 | 1441.6 | - | - | - | * | * | T | - | - | - |
| Nonanoic acid | 1230.0 | 2.80 | 1415.0 | - | - | - | - | - | - | - | * | P |
| Ornithine | 1885.0 | 3.16 | 1789.8 | - | - | - | - | - | - | - | * | T |
| Orotic acid | 1986.9 | 2.55 | 1854.5 | ** | * | C | * | * | C | - | - | - |
| Oxalacetic acid | 1600.0 | 2.78 | 1619.3 | - | - | - | - | - | - | - | * | T |
| Palmitoleic acid | 2348.3 | 2.83 | 2100.9 | *** | * | P | *** | * | T | - | - | - |
| Pentitol-1-desoxy | 1300.0 | 4.97 | 1454.0 | - | - | - | *** | * | T | - | * | T |
| Pentitol-3-desoxy | 1490.0 | 4.22 | 1557.9 | *** | * | P | *** | * | T | * | * | T |
| Phenylalanine | 1784.1 | 2.68 | 1728.4 | *** | * | P | *** | * | T | * | * | T |
| Phosphoric acid | 1085.1 | 2.75 | 1339.9 | ** | * | P | - | - | - | - | * | P |
| Pipecolic acid | 1130.0 | 3.10 | 1363.1 | *** | * | P | ** | * | T | * | * | P |
| Pseudouridine | 2715.0 | 3.08 | 2382.5 | - | - | - | ** | * | T | - | * | T |
| Pyroglutamic acid | 1715.2 | 2.29 | 1686.8 | ** | * | P | *** | * | T | - | * | T |
| Pyruvic acid | 784.9 | 1.96 | 1181.6 | *** | * | P | *** | * | T | - | * | P |
| Saccharide_178 | 1819.2 | 3.77 | 1750.3 | * | * | C | * | * | C | - | - | - |
| Saccharide_179 | 1976.7 | 3.54 | 1848.6 | ** | * | C | - | - | - | - | * | T |
| Saccharide_180 | 1795.0 | 4.49 | 1736.1 | ** | * | P | ** | * | T | - | - | - |
| Saccharide_181 | 2529.1 | 3.99 | 2237.0 | *** | * | C | * | * | C | * | * | T |
| Saccharide_184 | 2665.0 | 3.93 | 2343.1 | ** | * | C | ** | * | C | - | - | - |
| Saccharide_838 | 2605.6 | 3.69 | 2295.6 | - | - | - | - | - | - | - | * | P |
| Serine | 1070.0 | 2.60 | 1332.1 | * | * | P | - | - | - | * | * | P |
| Stearic acid | 2600.1 | 3.02 | 2290.9 | * | * | P | *** | * | T | - | * | T |
| Sugar alcohol_188 | 1909.1 | 3.82 | 1805.1 | *** | * | C | ** | * | C | - | * | T |
| Sugar alcohol_200 | 1890.0 | 0.66 | 1791.3 | - | - | - | - | - | - | - | * | T |
| Sugar alcohol_201 | 1915.0 | 0.49 | 1806.8 | - | - | - | * | * | T | - | * | T |
| Tartaric acid | 1680.9 | 3.20 | 1667.1 | - | - | - | - | - | - | * | * | T |
| Threonic acid | 1491.4 | 3.52 | 1558.3 | ** | * | C | - | - | - | - | * | T |
| Threonine | 1165.0 | 3.55 | 1381.3 | - | - | - | * | * | C | - | * | P |
| Tricarballylic acid | 1930.0 | 2.65 | 1817.8 | - | - | - | - | - | - | - | * | P |
| Tryptophan | 2720.0 | 2.57 | 2386.1 | *** | * | C | ** | * | C | - | - | - |
| Tyrosine | 2289.9 | 2.39 | 2058.8 | ** | * | C | *** | * | C | - | * | P |
| Uncertain ID_005 | 1805.0 | 3.03 | 1741.2 | * | * | P | - | - | - | - | * | P |
| Uncertain ID_018 | 1845.0 | 3.42 | 1765.7 | ** | * | C | * | * | C | - | - | - |
| Uncertain ID_025 | 1619.2 | 2.86 | 1630.7 | - | - | - | - | - | - | - | * | T |
| Uncertain ID_046 | 667.5 | 1.86 | 1116.3 | ** | * | P | - | - | - | - | * | P |
| Uncertain ID_050 | 1825.0 | 2.14 | 1752.8 | *** | * | P | *** | * | T | - | * | T |
| Uncertain ID_107 | 1536.2 | 2.20 | 1582.5 | * | * | C | - | - | - | - | * | T |
| Uncertain ID_118 | 2960.0 | 0.28 | 2587.2 | - | - | - | - | - | - | - | * | T |
| Uncertain ID_127 | 2933.3 | 0.26 | 2564.0 | - | - | - | - | - | - | - | * | T |
| Uncertain ID_151 | 2407.0 | 3.21 | 2144.6 | - | - | - | - | * | C | - | - | - |
| Uncertain ID_154 | 585.0 | 3.49 | 1063.2 | ** | * | P | - | - | - | - | * | P |
| Uncertain ID_168 | 1955.0 | 1.94 | 1833.5 | *** | * | P | *** | * | T | - | - | - |
| Uncertain ID_194 | 1415.0 | 2.71 | 1515.4 | * | * | P | ** | * | T | - | - | - |
| Uncertain ID_195 | 1320.0 | 2.12 | 1463.3 | ** | * | P | - | - | - | - | * | P |
| Uncertain ID_196 | 2010.9 | 3.59 | 1870.7 | *** | * | C | *** | * | C | - | - | - |
| Uncertain ID_199 | 1858.1 | 3.60 | 1773.8 | *** | * | C | *** | * | C | - | - | - |
| Uncertain ID_826 | 2418.0 | 3.57 | 2153.0 | - | - | - | * | * | C | - | - | - |
| Uncertain ID_840 | 1530.0 | 3.76 | 1579.9 | *** | * | C | ** | * | C | - | - | - |
| Uncertain ID_841 | 2020.0 | 4.42 | 1877.0 | * | * | C | ** | * | C | - | * | P |
| Uncertain ID_842 | 2209.3 | 3.40 | 2002.0 | *** | * | C | ** | * | C | - | - | - |
| Unknown_203 | 566.9 | 2.29 | 1049.4 | - | - | - | - | - | - | - | * | P |
| Unknown_205 | 1590.0 | 2.87 | 1613.5 | - | - | - | - | - | - | - | * | T |
| Unknown_206 | 510.2 | 2.25 | 1008.9 | - | - | - | - | - | - | - | * | P |
| Unknown_207 | 1764.9 | 2.69 | 1716.7 | - | - | - | - | - | - | - | * | P |
| Unknown_211 | 1346.1 | 2.51 | 1477.6 | - | - | - | - | - | - | * | * | P |
| Unknown_213 | 3674.8 | 2.02 | 3296.4 | *** | * | C | *** | * | C | - | * | P |
| Unknown_216 | 530.0 | 1.84 | 1022.7 | - | - | - | - | - | - | - | * | P |
| Unknown_218 | 1199.8 | 1.84 | 1398.3 | - | - | - | - | - | - | - | * | T |
| Unknown_222 | 1719.0 | 2.10 | 1688.9 | - | - | - | - | - | - | - | * | P |
| Unknown_224 | 760.0 | 2.84 | 1168.3 | *** | * | P | *** | * | T | - | * | P |
| Unknown_225 | 515.6 | 1.85 | 1012.4 | - | - | - | - | - | - | * | * | P |
| Unknown_229 | 1254.9 | 2.66 | 1428.4 | ** | * | C | - | - | - | - | * | T |
| Unknown_230 | 549.2 | 2.32 | 1036.8 | ** | * | P | - | - | - | ** | * | P |
| Unknown_231 | 1090.0 | 2.42 | 1342.3 | ** | * | P | - | - | - | * | * | P |
| Unknown_235 | 1349.5 | 2.82 | 1479.6 | * | * | C | - | - | - | - | * | T |
| Unknown_238 | 970.0 | 3.11 | 1280.6 | - | - | - | - | - | - | - | * | T |
| Unknown_242 | 1550.0 | 2.94 | 1590.5 | *** | * | C | * | * | C | * | * | T |
| Unknown_244 | 1645.1 | 3.02 | 1646.0 | - | - | - | * | * | T | - | * | T |
| Unknown_247 | 1770.1 | 3.13 | 1720.2 | * | * | P | ** | * | T | - | - | - |
| Unknown_248 | 1799.5 | 3.15 | 1738.0 | ** | * | P | *** | * | T | - | * | T |
| Unknown_249 | 1864.4 | 3.58 | 1777.6 | - | - | - | * | * | C | - | * | P |
| Unknown_250 | 1886.7 | 3.24 | 1790.9 | - | - | - | - | - | - | - | * | T |
| Unknown_252 | 2285.0 | 2.93 | 2055.7 | * | * | C | *** | * | C | - | * | P |
| Unknown_253 | 2445.4 | 2.69 | 2172.7 | *** | * | P | *** | * | T | - | * | T |
| Unknown_254 | 2765.0 | 2.91 | 2423.3 | ** | * | C | - | - | - | - | * | T |
| Unknown_255 | 1933.8 | 2.89 | 1820.5 | *** | * | C | * | * | C | - | * | T |
| Unknown_256 | 1105.4 | 3.80 | 1350.9 | - | - | - | - | - | - | - | * | P |
| Unknown_259 | 2690.3 | 2.48 | 2362.2 | *** | * | P | *** | * | T | - | - | - |
| Unknown_266 | 2260.0 | 2.19 | 2037.3 | *** | * | C | *** | * | C | - | - | - |
| Unknown_267 | 1055.1 | 2.36 | 1324.3 | - | - | - | - | - | - | * | * | P |
| Unknown_268 | 1895.0 | 3.64 | 1796.1 | - | - | - | * | * | T | * | * | T |
| Unknown_270 | 626.4 | 3.90 | 1093.1 | * | * | P | - | - | - | ** | * | P |
| Unknown_271 | 1169.3 | 3.03 | 1383.3 | * | * | P | - | - | - | - | - | - |
| Unknown_275 | 2000.8 | 3.79 | 1864.2 | ** | * | P | * | * | T | - | - | - |
| Unknown_280 | 2485.0 | 3.60 | 2202.8 | - | - | - | - | - | - | - | * | P |
| Unknown_281 | 680.0 | 3.38 | 1124.1 | ** | * | P | - | - | - | * | * | P |
| Unknown_284 | 586.7 | 2.00 | 1063.4 | * | * | P | - | - | - | - | * | P |
| Unknown_286 | 2005.9 | 3.56 | 1867.4 | ** | * | C | * | * | C | - | - | - |
| Unknown_288 | 945.4 | 2.43 | 1267.3 | ** | * | P | - | - | - | - | * | P |
| Unknown_292 | 499.6 | 1.62 | 1000.9 | - | - | - | - | - | - | - | * | T |
| Unknown_294 | 725.1 | 2.18 | 1148.5 | ** | * | P | - | - | - | * | * | P |
| Unknown_296 | 2457.7 | 3.71 | 2182.5 | ** | * | C | * | * | C | - | - | - |
| Unknown_301 | 2436.1 | 2.76 | 2165.8 | *** | * | C | *** | * | C | - | - | - |
| Unknown_302 | 666.3 | 1.99 | 1115.8 | ** | * | P | ** | * | T | - | * | P |
| Unknown_303 | 1900.0 | 2.57 | 1798.5 | ** | * | P | *** | * | T | * | * | T |
| Unknown_306 | 795.5 | 2.03 | 1187.5 | *** | * | P | *** | * | T | - | * | P |
| Unknown_308 | 545.3 | 2.12 | 1033.9 | - | - | - | * | * | C | - | * | P |
| Unknown_309 | 1525.2 | 1.80 | 1576.1 | * | * | C | * | * | C | - | - | - |
| Unknown_311 | 1825.0 | 2.33 | 1752.9 | - | - | - | ** | * | T | - | * | T |
| Unknown_313 | 1959.8 | 2.36 | 1836.8 | * | * | P | ** | * | T | - | - | - |
| Unknown_317 | 1180.0 | 2.46 | 1388.4 | - | - | - | - | - | - | - | * | T |
| Unknown_319 | 1330.0 | 2.81 | 1469.1 | - | * | P | - | - | - | - | - | - |
| Unknown_323 | 1748.2 | 2.28 | 1706.4 | - | - | - | * | * | T | - | * | T |
| Unknown_326 | 1320.0 | 2.66 | 1463.6 | - | - | - | - | - | - | - | * | T |
| Unknown_327 | 672.3 | 1.92 | 1119.0 | *** | * | P | *** | * | T | - | * | P |
| Unknown_328 | 2022.6 | 2.28 | 1877.3 | - | - | - | - | - | - | - | * | T |
| Unknown_330 | 2405.0 | 2.38 | 2142.5 | - | - | - | * | * | C | - | - | - |
| Unknown_334 | 2790.0 | 2.15 | 2443.5 | *** | * | P | *** | * | T | ** | * | T |
| Unknown_336 | 1275.2 | 2.94 | 1439.5 | - | - | - | * | * | C | - | * | P |
| Unknown_337 | 475.1 | 1.99 | 983.6 | ** | * | P | ** | * | T | - | - | - |
| Unknown_339 | 483.4 | 1.84 | 989.5 | - | - | - | - | - | - | - | * | P |
| Unknown_341 | 523.5 | 2.21 | 1018.4 | ** | * | P | - | - | - | * | * | P |
| Unknown_342 | 539.8 | 2.21 | 1030.0 | ** | * | P | - | - | - | - | - | - |
| Unknown_344 | 565.0 | 3.23 | 1048.7 | ** | * | P | - | - | - | - | * | P |
| Unknown_345 | 567.2 | 2.21 | 1049.6 | - | - | - | - | - | - | * | * | P |
| Unknown_347 | 595.0 | 2.47 | 1069.6 | ** | * | P | - | - | - | - | * | P |
| Unknown_351 | 644.5 | 2.42 | 1103.8 | * | * | P | - | - | - | - | * | P |
| Unknown_352 | 660.3 | 2.75 | 1112.8 | * | * | P | - | - | - | - | * | P |
| Unknown_353 | 670.0 | 3.11 | 1118.4 | * | * | P | - | - | - | - | - | - |
| Unknown_354 | 706.1 | 2.57 | 1138.1 | *** | * | P | *** | * | T | - | - | - |
| Unknown_358 | 725.0 | 1.95 | 1148.3 | - | - | - | - | - | - | - | * | P |
| Unknown_364 | 775.1 | 2.25 | 1176.3 | ** | * | P | - | - | - | * | * | P |
| Unknown_365 | 803.9 | 3.27 | 1192.9 | - | - | - | - | - | - | * | * | P |
| Unknown_373 | 922.8 | 2.90 | 1255.7 | * | * | P | - | - | - | - | * | P |
| Unknown_375 | 941.0 | 2.56 | 1265.0 | ** | * | P | - | - | - | - | * | P |
| Unknown_377 | 961.1 | 2.43 | 1275.6 | ** | * | P | - | - | - | *** | * | P |
| Unknown_379 | 970.0 | 2.45 | 1280.2 | - | - | - | - | - | - | - | * | P |
| Unknown_380 | 993.4 | 2.06 | 1292.4 | - | - | - | - | - | - | - | * | P |
| Unknown_382 | 999.9 | 4.00 | 1296.7 | - | - | - | - | - | - | * | * | P |
| Unknown_383 | 1001.6 | 2.27 | 1296.8 | - | - | - | - | - | - | - | * | P |
| Unknown_384 | 1010.1 | 2.48 | 1301.4 | ** | * | P | - | - | - | * | * | P |
| Unknown_385 | 1014.1 | 3.15 | 1303.8 | - | - | - | ** | * | C | - | * | P |
| Unknown_386 | 1039.6 | 2.56 | 1316.5 | *** | * | P | ** | * | T | - | - | - |
| Unknown_391 | 1115.1 | 2.57 | 1355.2 | - | - | - | - | - | - | - | * | P |
| Unknown_393 | 1125.0 | 2.21 | 1360.1 | ** | * | P | * | * | T | - | * | P |
| Unknown_397 | 1144.9 | 2.75 | 1370.6 | * | * | P | - | - | - | * | * | P |
| Unknown_401 | 1188.7 | 1.86 | 1392.6 | * | * | P | *** | * | T | - | * | T |
| Unknown_402 | 1194.4 | 2.01 | 1395.6 | - | - | - | * | * | T | - | * | T |
| Unknown_403 | 1195.0 | 3.39 | 1396.6 | - | - | - | - | - | - | - | * | T |
| Unknown_407 | 1230.0 | 3.29 | 1415.3 | - | - | - | - | - | - | - | * | P |
| Unknown_409 | 1235.0 | 2.72 | 1417.7 | - | - | - | - | - | - | - | * | P |
| Unknown_410 | 1235.0 | 4.07 | 1418.4 | - | - | - | * | * | C | - | - | - |
| Unknown_411 | 1240.0 | 2.53 | 1420.3 | * | * | P | - | - | - | - | * | P |
| Unknown_412 | 1245.0 | 2.80 | 1423.1 | - | - | - | ** | * | C | - | - | - |
| Unknown_414 | 1258.7 | 2.76 | 1430.5 | - | - | - | - | - | - | - | * | P |
| Unknown_421 | 1280.0 | 2.58 | 1441.9 | ** | * | C | * | * | C | - | - | - |
| Unknown_422 | 1295.0 | 3.44 | 1450.5 | * | * | C | - | - | - | - | * | T |
| Unknown_425 | 1309.8 | 2.48 | 1458.0 | - | - | - | * | * | C | - | - | - |
| Unknown_429 | 1350.0 | 2.69 | 1479.8 | - | - | - | ** | * | C | - | * | P |
| Unknown_430 | 1365.0 | 2.88 | 1488.0 | *** | * | C | *** | * | C | - | - | - |
| Unknown_431 | 1365.6 | 2.33 | 1488.0 | - | - | - | - | - | - | - | * | P |
| Unknown_433 | 1374.7 | 2.34 | 1493.0 | - | - | - | - | - | - | - | * | P |
| Unknown_435 | 1395.0 | 2.11 | 1504.0 | - | - | - | * | * | C | - | - | - |
| Unknown_436 | 1409.6 | 2.27 | 1512.2 | * | * | P | *** | * | T | - | * | T |
| Unknown_443 | 1435.8 | 2.57 | 1526.8 | * | * | C | - | - | - | - | * | T |
| Unknown_445 | 1455.0 | 1.94 | 1537.2 | - | - | - | * | * | C | - | * | P |
| Unknown_449 | 1470.0 | 2.66 | 1545.9 | * | * | C | - | - | - | - | * | T |
| Unknown_450 | 1475.0 | 2.26 | 1548.5 | * | * | C | - | - | - | - | * | T |
| Unknown_457 | 1510.0 | 2.82 | 1568.2 | - | - | - | - | - | - | - | * | T |
| Unknown_459 | 1520.1 | 3.72 | 1574.4 | * | * | C | - | - | - | - | - | - |
| Unknown_467 | 1550.4 | 2.92 | 1590.7 | *** | * | C | * | * | C | * | * | T |
| Unknown_468 | 1560.0 | 2.49 | 1595.8 | ** | * | C | - | - | - | - | * | T |
| Unknown_470 | 1570.0 | 4.02 | 1602.4 | * | * | C | - | - | - | * | * | T |
| Unknown_473 | 1590.0 | 2.67 | 1613.3 | - | - | - | - | - | - | - | * | T |
| Unknown_474 | 1593.8 | 2.55 | 1615.5 | - | - | - | - | - | - | - | * | T |
| Unknown_478 | 1614.9 | 2.68 | 1628.0 | *** | * | C | *** | * | C | - | - | - |
| Unknown_480 | 1620.3 | 3.01 | 1631.4 | - | - | - | - | - | - | - | * | T |
| Unknown_482 | 1625.0 | 2.84 | 1634.0 | - | - | - | - | - | - | - | * | T |
| Unknown_485 | 1640.5 | 2.41 | 1642.9 | *** | * | P | *** | * | T | - | - | - |
| Unknown_486 | 1646.4 | 4.41 | 1647.5 | ** | * | P | * | * | T | - | - | - |
| Unknown_490 | 1660.6 | 2.27 | 1654.6 | - | - | - | ** | * | T | * | * | T |
| Unknown_491 | 1662.9 | 3.00 | 1656.4 | *** | * | P | *** | * | T | - | * | T |
| Unknown_492 | 1670.0 | 2.39 | 1660.2 | *** | * | P | * | * | T | - | - | - |
| Unknown_493 | 1672.7 | 2.49 | 1661.9 | ** | * | P | * | * | T | - | - | - |
| Unknown_495 | 1695.0 | 3.26 | 1675.4 | *** | * | P | *** | * | T | * | * | T |
| Unknown_497 | 1704.9 | 2.71 | 1680.9 | *** | * | P | *** | * | T | - | * | T |
| Unknown_500 | 1731.8 | 2.43 | 1696.6 | ** | * | P | - | - | - | - | * | P |
| Unknown_501 | 1732.5 | 3.12 | 1697.5 | * | * | P | * | * | T | - | - | - |
| Unknown_502 | 1741.1 | 2.14 | 1702.0 | - | - | - | - | - | - | - | * | P |
| Unknown_505 | 1755.0 | 2.48 | 1710.6 | - | - | - | - | - | - | - | * | P |
| Unknown_506 | 1760.0 | 3.60 | 1714.3 | - | - | - | - | * | C | - | * | P |
| Unknown_511 | 1770.4 | 2.23 | 1719.8 | ** | * | P | * | * | T | - | - | - |
| Unknown_515 | 1790.0 | 2.44 | 1731.8 | - | - | - | - | - | - | - | * | T |
| Unknown_516 | 1799.6 | 3.57 | 1738.3 | ** | * | P | - | - | - | - | * | P |
| Unknown_518 | 1810.0 | 2.79 | 1744.1 | *** | * | P | *** | * | T | - | - | - |
| Unknown_521 | 1825.2 | 2.85 | 1753.3 | - | * | C | *** | * | C | - | * | P |
| Unknown_525 | 1855.1 | 2.72 | 1771.4 | * | * | P | * | * | T | - | - | - |
| Unknown_526 | 1860.5 | 2.87 | 1774.8 | - | - | - | ** | * | T | - | * | T |
| Unknown_527 | 1875.8 | 2.14 | 1783.6 | *** | * | P | ** | * | T | - | * | P |
| Unknown_529 | 1890.0 | 1.71 | 1791.9 | - | - | - | - | - | - | - | * | P |
| Unknown_530 | 1890.0 | 2.78 | 1792.6 | * | * | P | *** | * | T | - | - | - |
| Unknown_531 | 1895.0 | 2.44 | 1795.4 | - | - | - | * | * | C | - | * | P |
| Unknown_534 | 1924.9 | 2.66 | 1814.5 | - | - | - | * | * | C | - | * | P |
| Unknown_536 | 1935.0 | 2.46 | 1820.9 | - | - | - | ** | * | T | - | - | - |
| Unknown_537 | 1940.1 | 2.48 | 1824.3 | - | - | - | * | * | T | - | - | - |
| Unknown_540 | 1950.7 | 2.73 | 1831.2 | *** | * | P | *** | * | T | - | * | T |
| Unknown_541 | 1956.6 | 2.17 | 1834.7 | * | * | P | - | - | - | - | - | - |
| Unknown_543 | 1974.4 | 2.36 | 1846.3 | *** | * | P | *** | * | T | - | - | - |
| Unknown_544 | 1975.0 | 2.03 | 1846.5 | - | - | - | * | * | C | - | - | - |
| Unknown_547 | 1995.0 | 2.33 | 1859.6 | - | - | - | * | * | T | * | * | T |
| Unknown_558 | 2090.0 | 2.07 | 1921.4 | - | - | - | - | * | C | - | - | - |
| Unknown_559 | 2093.0 | 1.83 | 1923.2 | - | - | - | - | - | - | - | * | T |
| Unknown_560 | 2110.0 | 1.76 | 1934.5 | - | - | - | - | - | - | - | * | P |
| Unknown_564 | 2125.6 | 1.87 | 1945.0 | *** | * | P | *** | * | T | - | - | - |
| Unknown_568 | 2140.2 | 2.05 | 1954.9 | ** | * | P | * | * | T | - | - | - |
| Unknown_570 | 2149.8 | 1.90 | 1961.2 | ** | * | P | * | * | T | - | - | - |
| Unknown_575 | 2169.0 | 2.27 | 1974.1 | - | - | - | - | - | - | - | * | P |
| Unknown_579 | 2180.0 | 2.94 | 1982.0 | * | * | C | * | * | C | - | - | - |
| Unknown_580 | 2185.0 | 3.10 | 1985.4 | - | - | - | - | - | - | - | * | P |
| Unknown_590 | 2275.0 | 2.97 | 2048.6 | - | - | - | - | - | - | - | * | P |
| Unknown_592 | 2285.0 | 3.16 | 2055.8 | - | * | P | ** | * | T | - | * | T |
| Unknown_593 | 2295.0 | 4.49 | 2063.9 | * | * | P | - | - | - | - | * | P |
| Unknown_594 | 2303.2 | 3.15 | 2068.8 | * | * | P | - | - | - | - | * | P |
| Unknown_600 | 2325.0 | 2.74 | 2084.1 | *** | * | P | ** | * | T | - | * | P |
| Unknown_604 | 2349.5 | 3.27 | 2102.0 | * | * | P | - | - | - | * | * | P |
| Unknown_607 | 2374.9 | 2.48 | 2120.3 | - | - | - | - | - | - | - | * | P |
| Unknown_608 | 2380.0 | 2.59 | 2124.1 | - | * | P | - | - | - | - | - | - |
| Unknown_620 | 2460.1 | 2.37 | 2183.3 | ** | * | P | - | - | - | - | - | - |
| Unknown_621 | 2463.2 | 1.77 | 2185.1 | *** | * | P | ** | * | T | - | - | - |
| Unknown_623 | 2479.6 | 2.55 | 2197.9 | *** | * | P | *** | * | T | - | - | - |
| Unknown_624 | 2480.6 | 2.38 | 2198.5 | - | * | P | - | - | - | - | * | P |
| Unknown_625 | 2483.5 | 3.18 | 2201.3 | ** | * | C | * | * | C | - | - | - |
| Unknown_626 | 2484.5 | 4.01 | 2202.7 | *** | * | C | ** | * | C | - | * | T |
| Unknown_627 | 2500.0 | 2.02 | 2213.1 | - | - | - | - | - | - | - | * | T |
| Unknown_628 | 2500.0 | 2.74 | 2213.6 | - | - | - | ** | * | T | - | - | - |
| Unknown_629 | 2510.0 | 2.44 | 2221.1 | *** | * | P | * | * | T | - | * | P |
| Unknown_632 | 2538.5 | 2.54 | 2243.1 | *** | * | P | *** | * | T | - | - | - |
| Unknown_634 | 2555.0 | 2.15 | 2255.5 | *** | * | P | *** | * | T | - | - | - |
| Unknown_635 | 2555.0 | 2.84 | 2256.0 | ** | * | C | *** | * | C | - | * | P |
| Unknown_636 | 2560.0 | 2.06 | 2259.3 | - | - | - | - | - | - | - | * | P |
| Unknown_637 | 2560.7 | 3.99 | 2261.3 | *** | * | C | ** | * | C | * | * | T |
| Unknown_638 | 2561.3 | 2.67 | 2260.7 | - | - | - | ** | * | T | ** | * | T |
| Unknown_639 | 2565.1 | 3.27 | 2264.2 | *** | * | C | *** | * | C | - | * | T |
| Unknown_652 | 2660.0 | 2.45 | 2338.0 | *** | * | P | *** | * | T | - | * | P |
| Unknown_653 | 2669.7 | 2.55 | 2345.8 | * | * | P | * | * | T | - | - | - |
| Unknown_654 | 2670.0 | 2.73 | 2346.2 | - | - | - | - | - | - | - | * | P |
| Unknown_655 | 2685.1 | 2.34 | 2358.0 | *** | * | P | *** | * | T | - | - | - |
| Unknown_656 | 2690.0 | 2.20 | 2361.8 | *** | * | P | * | * | T | - | - | - |
| Unknown_657 | 2691.0 | 3.00 | 2363.2 | * | * | P | ** | * | T | - | - | - |
| Unknown_658 | 2715.0 | 2.24 | 2381.8 | - | - | - | - | - | - | - | * | T |
| Unknown_659 | 2720.4 | 2.11 | 2386.0 | - | - | - | ** | * | T | - | * | T |
| Unknown_660 | 2725.0 | 2.02 | 2389.6 | ** | * | P | ** | * | T | - | - | - |
| Unknown_661 | 2740.0 | 2.47 | 2402.1 | - | - | - | - | - | - | - | * | P |
| Unknown_662 | 2752.3 | 1.88 | 2411.9 | - | - | - | * | * | T | - | * | T |
| Unknown_667 | 2790.0 | 4.87 | 2445.7 | ** | * | P | * | * | T | - | * | P |
| Unknown_668 | 2825.0 | 2.67 | 2473.1 | *** | * | P | *** | * | T | - | * | P |
| Unknown_670 | 2831.3 | 2.12 | 2477.9 | - | - | - | - | - | - | - | * | T |
| Unknown_672 | 2850.0 | 3.22 | 2494.3 | *** | * | P | *** | * | T | - | * | P |
| Unknown_675 | 2870.0 | 2.55 | 2510.9 | ** | * | P | *** | * | T | - | - | - |
| Unknown_676 | 2870.0 | 3.28 | 2511.6 | *** | * | C | ** | * | C | * | * | T |
| Unknown_677 | 2885.4 | 2.61 | 2524.3 | *** | * | P | *** | * | T | - | * | P |
| Unknown_678 | 2889.7 | 2.92 | 2528.3 | *** | * | P | ** | * | T | - | - | - |
| Unknown_679 | 2897.3 | 1.98 | 2534.1 | * | * | P | ** | * | T | - | - | - |
| Unknown_680 | 2925.8 | 2.97 | 2559.8 | ** | * | P | * | * | T | - | - | - |
| Unknown_681 | 2938.1 | 2.75 | 2570.3 | *** | * | C | - | - | - | ** | * | T |
| Unknown_685 | 2969.1 | 2.05 | 2596.6 | ** | * | C | ** | * | C | - | - | - |
| Unknown_687 | 2982.1 | 3.69 | 2609.8 | - | - | - | ** | * | T | - | - | - |
| Unknown_688 | 2985.0 | 1.80 | 2610.7 | *** | * | C | ** | * | C | - | - | - |
| Unknown_690 | 2987.2 | 2.49 | 2613.4 | *** | * | P | *** | * | T | - | - | - |
| Unknown_693 | 3025.0 | 2.88 | 2648.1 | *** | * | P | *** | * | T | - | - | - |
| Unknown_694 | 3044.9 | 2.67 | 2666.0 | * | * | P | * | * | T | - | - | - |
| Unknown_696 | 3075.7 | 3.37 | 2694.6 | - | - | - | - | - | - | - | * | T |
| Unknown_697 | 3099.6 | 2.55 | 2715.6 | *** | * | P | *** | * | T | - | - | - |
| Unknown_701 | 3171.4 | 2.42 | 2780.7 | * | * | P | * | * | T | - | - | - |
| Unknown_704 | 3295.0 | 2.53 | 2902.5 | * | * | C | ** | * | C | - | * | P |
| Unknown_705 | 3445.0 | 2.26 | 3052.3 | - | - | - | - | - | - | - | * | P |
| Unknown_707 | 3590.2 | 1.85 | 3202.4 | ** | * | C | *** | * | C | - | * | P |
| Unknown_708 | 3606.2 | 1.99 | 3220.3 | - | - | - | - | - | - | - | * | T |
| Unknown_709 | 3699.7 | 0.92 | 3322.9 | * | * | C | - | - | - | - | * | T |
| Unknown_710 | 3990.0 | 4.26 | 3649.2 | *** | * | C | *** | * | C | - | - | - |
| Unknown_711 | 1829.9 | 3.78 | 1756.7 | - | - | - | - | - | - | * | * | T |
| Unknown_717 | 2106.2 | 3.24 | 1933.0 | *** | * | C | *** | * | C | - | - | - |
| Unknown_718 | 854.0 | 2.45 | 1219.2 | * | * | P | - | - | - | - | * | P |
| Unknown_731 | 1991.1 | 2.36 | 1857.1 | ** | * | P | *** | * | T | - | * | T |
| Unknown_735 | 2810.0 | 2.04 | 2460.0 | *** | * | P | *** | * | T | - | * | T |
| Unknown_736 | 2913.6 | 1.99 | 2548.3 | *** | * | P | *** | * | T | - | * | T |
| Unknown_737 | 2930.2 | 2.23 | 2563.0 | *** | * | P | *** | * | T | - | * | T |
| Unknown_738 | 1340.4 | 2.67 | 1474.6 | *** | * | C | *** | * | C | - | - | - |
| Unknown_742 | 490.0 | 2.39 | 994.6 | *** | * | P | *** | * | T | - | * | P |
| Unknown_743 | 545.2 | 2.05 | 1033.7 | - | - | - | - | - | - | - | * | P |
| Unknown_745 | 770.0 | 3.17 | 1174.0 | ** | * | P | - | - | - | * | * | P |
| Unknown_747 | 1245.2 | 3.15 | 1423.4 | - | - | - | *** | * | C | - | * | P |
| Unknown_748 | 1745.0 | 2.89 | 1704.8 | ** | * | P | * | * | T | - | - | - |
| Unknown_749 | 705.7 | 2.45 | 1137.9 | - | - | - | - | - | - | - | * | P |
| Unknown_751 | 2710.0 | 2.45 | 2378.0 | - | - | - | - | - | - | - | * | P |
| Unknown_752 | 1400.1 | 2.88 | 1507.2 | * | * | P | - | - | - | - | - | - |
| Unknown_754 | 1005.4 | 2.23 | 1298.7 | - | - | - | - | - | - | - | * | P |
| Unknown_755 | 1012.6 | 2.23 | 1302.5 | - | - | - | - | - | - | - | * | P |
| Unknown_757 | 2060.5 | 1.68 | 1901.5 | - | - | - | - | - | - | - | * | P |
| Unknown_760 | 1325.1 | 2.43 | 1466.2 | - | - | - | - | - | - | - | * | T |
| Unknown_762 | 1688.0 | 2.29 | 1670.7 | * | * | P | * | * | T | - | - | - |
| Unknown_765 | 1364.5 | 2.64 | 1487.6 | - | - | - | - | - | - | - | * | P |
| Unknown_766 | 1374.6 | 2.54 | 1493.1 | - | - | - | - | - | - | - | * | P |
| Unknown_768 | 1465.0 | 2.04 | 1542.8 | *** | * | C | * | * | C | - | * | T |
| Unknown_770 | 1880.1 | 2.70 | 1786.5 | - | - | - | ** | * | C | - | * | P |
| Unknown_771 | 1976.1 | 1.86 | 1847.1 | * | * | C | - | - | - | - | - | - |
| Unknown_777 | 2230.0 | 0.46 | 2014.6 | *** | * | C | *** | * | C | - | - | - |
| Unknown_778 | 2394.9 | 2.45 | 2135.1 | ** | * | P | ** | * | T | - | - | - |
| Unknown_780 | 2740.2 | 3.13 | 2402.8 | *** | * | C | *** | * | C | - | - | - |
| Unknown_781 | 2761.5 | 2.03 | 2419.6 | - | - | - | - | - | - | - | * | P |
| Unknown_784 | 3105.0 | 4.80 | 2722.5 | *** | * | C | ** | * | C | - | * | T |
| Unknown_785 | 1187.2 | 1.80 | 1391.8 | * | * | P | *** | * | T | - | * | T |
| Unknown_786 | 2049.2 | 3.71 | 1895.4 | ** | * | C | - | - | - | - | - | - |
| Unknown_789 | 2535.0 | 2.58 | 2240.4 | *** | * | P | *** | * | T | - | * | P |
| Unknown_790 | 2720.0 | 4.61 | 2387.7 | - | - | - | - | - | - | * | * | T |
| Unknown_793 | 2545.0 | 3.21 | 2248.6 | *** | * | C | ** | * | C | - | - | - |
| Unknown_798 | 855.0 | 2.36 | 1219.7 | ** | * | P | - | - | - | ** | * | P |
| Unknown_801 | 544.0 | 2.57 | 1033.3 | * | * | P | - | - | - | - | - | - |
| Unknown_802 | 2059.8 | 3.25 | 1902.1 | *** | * | C | *** | * | C | - | - | - |
| Unknown_805 | 2492.1 | 3.27 | 2208.0 | - | - | - | * | * | T | - | * | T |
| Unknown_806 | 1917.6 | 2.14 | 1809.5 | * | * | P | - | - | - | - | - | - |
| Unknown_808 | 544.9 | 1.96 | 1033.5 | * | * | P | - | - | - | - | - | - |
| Unknown_809 | 1198.4 | 3.56 | 1398.5 | ** | * | P | - | - | - | - | * | P |
| Unknown_810 | 2160.4 | 3.40 | 1969.2 | *** | * | C | *** | * | C | - | - | - |
| Unknown_811 | 1445.0 | 2.93 | 1532.2 | * | * | P | *** | * | T | * | * | T |
| Unknown_816 | 2815.2 | 4.49 | 2466.4 | ** | * | C | - | - | - | - | * | T |
| Unknown_817 | 2017.3 | 2.20 | 1873.9 | ** | * | P | - | - | - | - | * | P |
| Unknown_844 | 1480.0 | 3.62 | 1552.0 | *** | * | C | *** | * | C | - | - | - |
| Unknown_847 | 1031.4 | 2.94 | 1312.5 | * | * | P | ** | * | T | - | - | - |
| Unknown_848 | 2345.7 | 3.17 | 2099.2 | * | * | P | *** | * | T | - | * | T |
| Unknown_850 | 3235.0 | 2.69 | 2842.7 | - | - | - | *** | * | T | - | * | T |
| Unknown_852 | 1577.1 | 2.08 | 1605.4 | - | - | - | - | - | - | - | * | T |
| Unknown_853 | 1971.8 | 2.93 | 1845.0 | - | - | - | - | - | - | - | * | T |
| Unknown_855 | 975.1 | 2.52 | 1282.9 | * | * | P | * | * | T | - | - | - |
| Unknown_856 | 509.9 | 2.66 | 1009.0 | * | * | P | - | - | - | - | * | P |
| Unknown_857 | 1943.3 | 3.18 | 1826.7 | * | * | C | ** | * | C | - | - | - |
| Unknown_861 | 643.0 | 3.78 | 1103.8 | * | * | P | - | - | - | - | * | P |
| Unknown_862 | 880.1 | 2.47 | 1233.0 | - | - | - | - | - | - | - | * | P |
| Unknown_863 | 940.0 | 2.11 | 1264.3 | ** | * | P | * | * | T | - | * | P |
| Unknown_864 | 1009.5 | 2.60 | 1301.1 | - | - | - | - | - | - | - | * | P |
| Unknown_865 | 1195.3 | 2.88 | 1396.5 | * | * | P | ** | * | T | - | - | - |
| Unknown_867 | 1326.1 | 3.23 | 1467.2 | *** | * | C | *** | * | C | - | - | - |
| Unknown_868 | 1410.0 | 1.99 | 1512.2 | - | - | - | * | * | T | - | * | T |
| Unknown_869 | 1625.0 | 3.12 | 1634.2 | - | - | - | - | - | - | - | * | P |
| Unknown_871 | 1700.7 | 2.71 | 1678.5 | ** | * | P | ** | * | T | - | - | - |
| Unknown_877 | 1862.6 | 1.91 | 1775.5 | - | - | - | - | - | - | - | * | P |
| Unknown_880 | 1991.0 | 2.69 | 1857.2 | - | - | - | * | * | C | - | * | P |
| Unknown_882 | 2074.6 | 2.41 | 1911.3 | *** | * | P | *** | * | T | - | - | - |
| Unknown_883 | 2105.0 | 2.47 | 1931.6 | ** | * | P | *** | * | T | - | - | - |
| Unknown_886 | 2148.8 | 3.01 | 1961.2 | - | * | P | - | - | - | - | - | - |
| Unknown_887 | 2275.3 | 2.32 | 2048.3 | * | * | C | - | * | C | - | * | T |
| Unknown_889 | 2330.0 | 2.05 | 2087.2 | - | - | - | - | - | - | - | * | P |
| Unknown_890 | 2350.0 | 2.52 | 2101.9 | * | * | C | * | * | C | - | - | - |
| Unknown_892 | 2420.0 | 2.14 | 2153.4 | - | - | - | * | * | C | - | * | P |
| Unknown_893 | 2430.0 | 2.04 | 2160.8 | - | - | - | - | - | - | - | * | P |
| Unknown_894 | 2455.0 | 1.95 | 2179.2 | ** | * | P | ** | * | T | - | - | - |
| Unknown_896 | 2475.0 | 4.84 | 2196.2 | *** | * | C | *** | * | C | - | * | T |
| Unknown_898 | 2480.9 | 2.76 | 2199.0 | * | * | C | - | - | - | - | - | - |
| Unknown_900 | 2524.8 | 2.53 | 2232.5 | - | - | - | - | - | - | - | * | P |
| Unknown_901 | 2605.0 | 2.22 | 2294.0 | - | - | - | * | * | C | - | - | - |
| Unknown_902 | 2609.5 | 4.14 | 2299.0 | *** | * | C | *** | * | C | - | - | - |
| Unknown_903 | 2610.0 | 2.57 | 2298.1 | * | * | C | ** | * | C | - | - | - |
| Unknown_906 | 2774.4 | 2.41 | 2430.7 | - | - | - | - | - | - | - | * | P |
| Unknown_907 | 2824.1 | 3.86 | 2473.3 | *** | * | C | ** | * | C | - | - | - |
| Unknown_909 | 2850.0 | 3.00 | 2494.2 | *** | * | C | *** | * | C | - | - | - |
| Unknown_910 | 2871.5 | 4.96 | 2514.3 | * | * | C | - | * | C | - | - | - |
| Unknown_911 | 3040.9 | 0.12 | 2660.0 | - | - | - | - | - | - | - | * | P |
| Unknown_912 | 3145.0 | 2.41 | 2756.7 | - | - | - | - | - | - | - | * | P |
| Unknown_914 | 3194.9 | 2.61 | 2802.5 | - | - | - | ** | * | C | * | * | P |
| Unknown_917 | 1961.7 | 2.12 | 1838.0 | ** | * | P | *** | * | T | - | - | - |
| Unknown_918 | 2280.0 | 3.39 | 2052.4 | * | * | P | ** | * | T | - | * | T |
| Unknown_920 | 2018.2 | 1.71 | 1874.1 | - | - | - | - | - | - | - | * | T |
| Unknown_922 | 476.3 | 1.79 | 984.4 | - | - | - | - | - | - | - | * | P |
| Unknown_923 | 1989.0 | 1.39 | 1855.1 | * | * | P | * | * | T | - | - | - |
| Unknown_925 | 1533.3 | 1.93 | 1580.7 | * | * | P | ** | * | T | - | - | - |
| Unknown_938 | 2922.9 | 2.86 | 2557.2 | *** | * | P | *** | * | T | - | - | - |
| Unknown_939 | 1280.0 | 3.23 | 1442.3 | * | * | P | ** | * | T | - | - | - |
| Unknown_940 | 1609.1 | 2.70 | 1624.6 | *** | * | P | ** | * | T | - | - | - |
| Unknown_941 | 1804.7 | 1.91 | 1740.4 | - | - | - | - | - | - | - | * | T |
| Unknown_942 | 1850.0 | 2.56 | 1768.2 | *** | * | P | *** | * | T | - | - | - |
| Unknown_943 | 2050.0 | 2.73 | 1895.3 | *** | * | P | *** | * | T | - | * | T |
| Unknown_944 | 2075.0 | 2.37 | 1911.6 | *** | * | P | *** | * | T | - | - | - |
| Unknown_946 | 2410.0 | 1.64 | 2145.7 | * | * | P | ** | * | T | - | - | - |
| Unknown_947 | 2510.0 | 2.02 | 2220.8 | *** | * | P | *** | * | T | - | * | T |
| Unknown_949 | 2661.8 | 2.07 | 2339.1 | *** | * | P | *** | * | T | * | * | T |
| Unknown_950 | 2685.0 | 1.93 | 2357.5 | - | * | P | * | * | T | - | - | - |
| Unknown_951 | 2894.6 | 1.48 | 2531.4 | *** | * | P | *** | * | T | - | - | - |
| Unknown_952 | 2906.1 | 1.74 | 2541.6 | *** | * | P | *** | * | T | - | - | - |
| Unknown_953 | 2918.8 | 2.90 | 2553.7 | *** | * | P | *** | * | T | - | * | T |
| Unknown_954 | 2922.3 | 3.38 | 2557.1 | *** | * | P | *** | * | T | - | - | - |
| Unknown_955 | 3010.0 | 2.83 | 2634.4 | *** | * | P | *** | * | T | - | - | - |
| Unknown_956 | 3025.0 | 1.90 | 2647.2 | *** | * | P | *** | * | T | - | - | - |
| Unknown_957 | 3058.7 | 2.21 | 2678.1 | *** | * | P | *** | * | T | - | * | T |
| Unknown_958 | 3156.5 | 3.02 | 2767.7 | *** | * | P | *** | * | T | - | - | - |
| Unknown_959 | 3184.8 | 2.48 | 2793.0 | *** | * | P | *** | * | T | - | - | - |
| Unknown_960 | 3692.8 | 2.39 | 3316.9 | ** | * | P | *** | * | T | - | * | T |
| Unknown_961 | 2065.4 | 2.71 | 1905.4 | * | * | P | *** | * | T | * | * | T |
| Unknown_962 | 3149.6 | 2.71 | 2761.2 | - | - | - | * | * | T | - | * | T |
| Unknown_963 | 1045.1 | 2.32 | 1319.1 | *** | * | P | *** | * | T | * | * | P |
| Unknown_964 | 1330.0 | 1.90 | 1468.6 | *** | * | P | *** | * | T | - | * | T |
| Unknown_967 | 1525.0 | 3.96 | 1577.2 | *** | * | C | ** | * | C | - | - | - |
| Unknown_968 | 1570.0 | 2.84 | 1601.7 | ** | * | C | ** | * | C | - | - | - |
| Unknown_969 | 1579.5 | 1.63 | 1606.5 | - | - | - | * | * | T | - | - | - |
| Unknown_970 | 1620.0 | 2.56 | 1630.9 | - | - | - | * | * | T | - | * | T |
| Unknown_971 | 1750.2 | 3.11 | 1708.1 | *** | * | P | *** | * | T | - | * | T |
| Unknown_972 | 1933.5 | 3.57 | 1820.7 | ** | * | P | *** | * | T | - | * | T |
| Unknown_973 | 2040.0 | 2.49 | 1888.7 | ** | * | P | * | * | T | - | - | - |
| Unknown_974 | 2065.0 | 2.96 | 1905.3 | * | * | P | ** | * | T | - | - | - |
| Unknown_976 | 2265.0 | 2.43 | 2041.0 | *** | * | P | *** | * | T | - | * | T |
| Unknown_979 | 2515.4 | 3.05 | 2225.7 | * | * | P | ** | * | T | - | * | T |
| Unknown_981 | 2748.2 | 2.05 | 2408.5 | *** | * | P | *** | * | T | * | * | T |
| Unknown_984 | 1715.0 | 3.26 | 1687.2 | *** | * | P | *** | * | T | - | - | - |
| Uracil | 1320.0 | 2.32 | 1463.4 | ** | * | P | *** | * | T | - | - | - |
| Urea | 1155.4 | 2.31 | 1375.7 | - | - | - | ** | * | T | - | * | T |
| Uric acid | 2560.1 | 2.49 | 2259.7 | - | - | - | - | - | - | * | * | T |
| Valine | 865.0 | 3.20 | 1225.4 | * | * | P | - | - | - | - | * | P |
| Xanthine | 2585.1 | 2.09 | 2278.5 | *** | * | P | *** | * | T | - | * | T |

^a^ Metabolites statistically significant in any of the pairwise comparisons (Para-A vs. control (P vs. C), Typhi vs. control (T vs. C) and Para-A vs. Typhi (P vs T)) according to covariance loadings w* (<|0.03|) and/or univariate p-values (≤ 0.05)

^b^ RT1 refers to the 1^st^ dimension retention time (s), RT2 refers to the 2^nd^ dimension retention time (s), RI1 refers to the 1^st^ dimension retention index

^c^ For each of the pairwise comparisons (Para-A vs. control, Typhi vs. control and Para-A vs. Typhi) the univariate p-value (p), the model covariance loadings (w*) and the change in concentration (c) for each metabolite is given. p-values are indicated by the degree of significance with *** for p ≤ 0.001, ** for p ≤ 0.01, * for p ≤ 0.05 and “–“ for p > 0.05 (indicating a non-significant p-value). For covariance loadings, w*, significance is indicated by * (<|0.03|) and non-significance by “–“. Change in metabolite concentration is indicated by a P for metabolites having higher concentration in Para-A samples, a T for metabolites having higher concentration in Typhi samples and a C for metabolites having higher concentration in control samples. A metabolite having a non-significant p-value as well as a non-significant model covariance loading value is not considered significant in that comparison.
